# Pro- vs. Anti-Inflammatory Features of Monocyte Subsets in Glioma Patients

**DOI:** 10.3390/ijms24031879

**Published:** 2023-01-18

**Authors:** Natalia Lehman, Wioleta Kowalska, Michał Zarobkiewicz, Marek Mazurek, Karolina Mrozowska, Agnieszka Bojarska-Junak, Radosław Rola

**Affiliations:** 1 Department of Clinical Immunology, Medical University of Lublin, 20-093 Lublin, Poland; 2 Department of Neurosurgery and Paediatric Neurosurgery, Medical University of Lublin, 20-093 Lublin, Poland

**Keywords:** glioma, glioblastoma, monocytes, SLAN, immune checkpoints, cytokines

## Abstract

Monocytes constitute a heterogenous group of antigen-presenting cells that can be subdivided based on CD14, CD16 and SLAN expression. This division reflects the functional diversity of cells that may play different roles in a variety of pathologies including gliomas. In the current study, the three monocyte subpopulations: classical (CD14^+^ CD16^+^ SLAN^−^), intermediate (CD14^dim^ CD16^+^ SLAN^−^) and non-classical (CD14^low/−^ CD16^+^ SLAN^+^) in glioma patients’ peripheral blood were analysed with flow cytometry. The immune checkpoint molecule (PD-1, PD-L1, SIRPalpha, TIM-3) expression along with pro- and anti-inflammatory cytokines (TNF, IL-12, TGF-beta, IL-10) were assessed. The significant overproduction of anti-inflammatory cytokines by intermediate monocytes was observed. Additionally, SLAN-positive cells overexpressed IL-12 and TNF when compared to the other two groups of monocytes. In conclusion, these results show the presence of different profiles of glioma patient monocytes depending on CD14, CD16 and SLAN expression. The bifold function of monocyte subpopulations might be an additional obstacle to the effectiveness of possible immunotherapies.

## 1. Introduction

Gliomas constitute a heterogenous group of tumors arising within the central nervous system (CNS) [1,2]. They differ from each other in biological properties, and thus various treatment strategies are used [3]. Glioblastoma multiforme (GBM) (WHO IV grade) is the most common malignant primary brain tumour [2]. Despite the use of various medical interventions, e.g., adjuvant radiation therapy, temozolomide administration (TMZ) [3], patients surviving over 3 years after diagnosis, called long-time survivors, constitute only 3–26% of all cases [4,5]. Currently, most of the research focuses on molecular aspects of the tumour glial cells, with special emphasis on vaccines specific for tumor cell antigens, which activate an antitumor immune response [6]. Currently, the most widely used prognostic factor in GBM is the methylation status of O-6-methylguanine-DNA methyltransferase (*MGMT)* gene promoter [7] *MGMT* promoter methylation silences its expression and is associated with longer overall survival in GBM patients, for example, as predictive factor of response to the TMZ treatment. [8]. The development of new treatment methods including immunotherapy requires an in-depth understanding of the tumour microenvironment and immune system cells.

Monocytes have a diverse role in inflammation and infection. They also play a role in cancerogenesis, formation of metastases [9] and risk of cancer-associated venous thrombosis [9,10,11]. Monocytes are commonly divided into three subsets based on CD14 and CD16 expression: classical monocytes (CD14^+^ CD16^−^), intermediate monocytes (CD14^+^ CD16^+^) and non-classical monocytes (CD14^low/−^ CD16^+^) [12,13]. The more accurate distinction of intermediate and non-classical monocytes is possible by the analysis of the 6-sulfo LacNac (SLAN) expression [14]. Thus, the current study defines monocytes as classical (CD14^+^ CD16^−^ SLAN^−^), intermediate (CD14^+^ CD16^+^ SLAN^−^) and non-classical (CD14^low/−^ CD16^+^ SLAN^+^) monocytes. Classical monocytes are characterised by the high expression of CCR2 (C-C motif chemokine receptor 2) and low CCR5 (C-C motif chemokine receptor 5) and CX3CR1 (chemokine (C-X3-C motif) receptor 1). On the contrary, CD16-positive monocytes are CCR2^low^ or negative, while intermediate monocytes have high levels of CCR5, and non-classical monocytes are CX3CR1-positive cells [15].

This division is reflected in the functional differentiation of monocytes and is potentially clinically relevant. Contrary to classical and intermediate monocytes which represent proinflammatory patterns, non-classical monocytes show increased expression of molecules such as PD-L1 (programmed cell death 1 ligand), PD-L2, Arginase1, IDO (Indoleamine-pyrrole 2,3-dioxygenase), and CD163; this suggests their immunosuppressive potential in the course of malignant neoplasms [16,17]. Additionally, murine studies suggest the involvement of CCL2-recruited Ly6C^+^ (lymphocyte antigen 6 complex, locus C1) inflammatory monocytes in bone metastases [18]. The inhibition of the CCL2–CCR2 pathway leads to diminished migration of classical monocytes, which resulted in a reduction of metastasis formation in vivo [19]. The recent studies present that glioblastoma cells also express CCR5 [20]. Moreover, CCL5 along with CCL2 secreted by GBM attracts various types of effector cells forming the tumor microenvironment, including myeloid cells [20,21]. In malignant gliomas, circulating monocytes migrate to tumor milieu and differentiate into glioma-associated microglia/macrophages (GAMs). GAMs mainly consist of bone-marrow-derived macrophages and monocytes cells (BDMCs) and resident microglia [22]. BDMC infiltration into glioblastoma tissues has been shown to be sustained by circulating monocytes [23]. That unique subset of monocytes might be potentially used as an additional agent in immunotherapies targeted to glioma cells. What is interesting is that both anti- and pro-carcinogenesis properties of GAMs were described [24]. Moreover, van den Bossche et al. distinguish a different pattern of glial fibrillary acidic protein (GFAP)-carrying monocytes in glioma patients compared to healthy volunteers [25]. Their finding suggests that an increased level of GFAP^+^ CD16^+^ monocytes is connected with possible brain tissue damage, e.g., stroke, or brain tumor.

The tumor microenvironment, including monocytes and GAMs, is attracting more and more attention as a possible target for immunotherapies in gliomas [26]. However, the role of monocyte subpopulations in glioma still remains unclear. Therefore, the major objective of this study was to characterize three subsets of monocytes from glioma patients’ peripheral blood (PB), with particular emphasis on immune checkpoints (PD-1 (programmed cell death 1 protein), PD-L1, SIRPalpha (signal-regulatory protein alpha), TIM-3 (T cell Ig and mucin domain 3), chemokine receptors (CCR2, CCR5, CX3CR1) expression, as well as pro- (TNF, IL-12) and anti-inflammatory (TGF-beta, IL-10) cytokines’ intracellular profile.

## 2. Results

### 2.1. The Reduction of Classical and Non-Classical Monocytes in Glioma Patients

**Figure 1:**
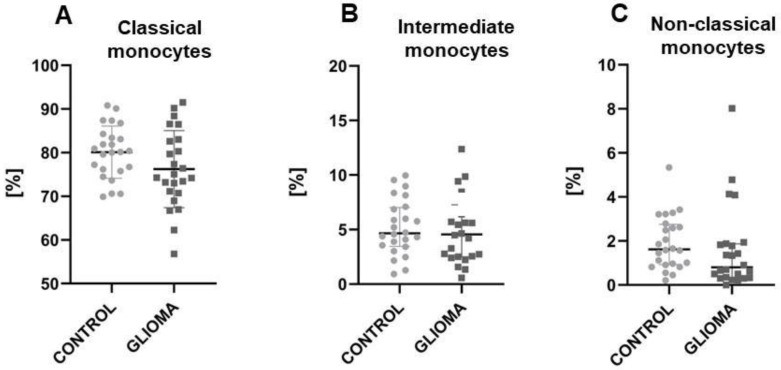
The results for the three monocyte subpopulations’ frequency based on the CD14, CD16 and SLAN expression. The comparison between glioma patients and the control group showed less numerous representations of classical and non-classical glioma patients’ monocytes. Unpaired *t*-student was used for graph A. In other cases U Mann-Whitney was used.

As expected, classical monocytes dominated in both glioma patients and healthy volunteers while non-classical monocytes were only the minor subset (Figure 1). Next, we analysed the expression of receptors for chemokines (CCR2, CCR5, CX3CR1). The highest expression of CCR2 was noted in classical (*p* < 0.0001), while higher expression of CCR5 was characteristic for intermediate (*p* < 0.0001). CX3CR1 expression was a hallmark of the non-classical subpopulation. Importantly, chemokine receptors characteristic of the given subset had higher expression in glioma patients than in the control group.

When comparing glioma patients to healthy volunteers, the reduction of classical and non-classical monocyte subpopulations in the study group was noticeable (Figure 1A,C). What is interesting is that a similar percentage of intermediate monocytes (approximately 5%) was noted in both glioma patients and healthy volunteers. (Figure 1B).

### 2.2. A Higher Expression of Immune Checkpoints from the PD-1/PD-L1 Pathway

**Figure 2:**
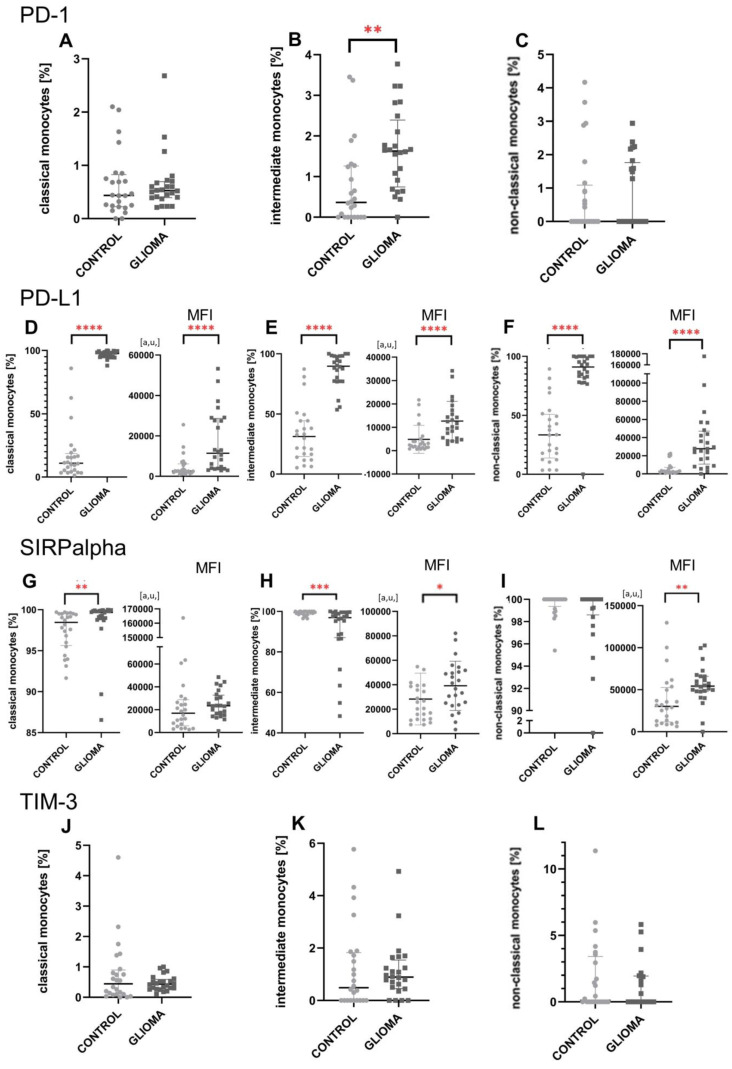
The results for immune checkpoint molecules expression on glioma patients’ monocytes vs. healthy individuals. Percentage of classical (**A**), intermediate (**B**) and non-classical (**C**) monocytes with PD-1 expression. As a consequence of the expression exceeding 95% for the PD-L1 (**D**–**F**) and SIRPalpha (**G**–**I**), the MFI was evaluated. Percentage of classical (**J**), intermediate (**K**) and non-classical (**L**) monocytes with TIM-3 expression. In all presented analyses, the U Mann-Whitney test was used. * *p* < 0.05, ** *p* < 0.01, *** *p* < 0.001, **** *p* < 0.0001. PD-1, programmed cell death 1 protein; PD-L1, programmed cell death 1 ligand; MFI, mean fluorescence intensity; SIRPalpha, signal-regulatory protein alpha.

**Figure 3:**
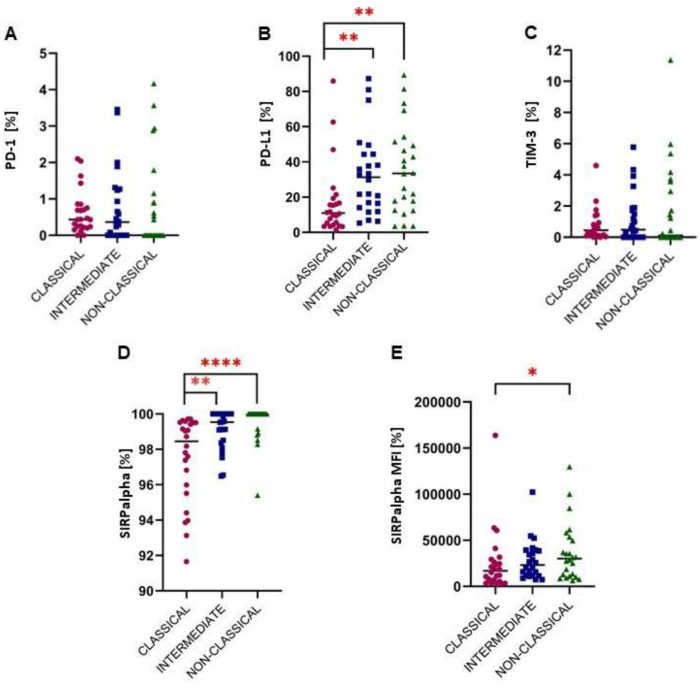
The identification of the differences in the immune checkpoint molecules: PD-1 (**A**) PD-L1 (**B**), TIM-3 (**C**) and SIRPalpha (**D,E**) expression between classical, intermediate and non-classical monocytes. Due to the expression exceeding 95% (**D**), for SIRPalpha the MFI assessment was used (**E**). * *p* < 0.05, ** *p* < 0.01, **** *p* < 0.0001 PD-1, programmed cell death 1 protein; PD-L1, programmed cell death 1 ligand; MFI, mean fluorescence intensity; SIRPalpha, signal-regulatory protein alpha.

The higher expression of PD-1 by all three subpopulations of monocytes was observed. Intermediate in particular cells were characterized by significant over-expression of PD-1 (*p* < 0.01) when compared to healthy individuals (Figure 2B). Non-classical monocytes had the lowest expression of PD-1 when compared to the classical and intermediate (Figure 3A). Significant overexpression of PD-L1 was noted in classical (*p* < 0.0001) intermediate (*p* < 0.0001) and non-classical (*p* < 0.0001) monocytes in gliomas patients (Figure 2D–F). Contrary to PD-1, intermediate and non-classical monocytes have a significantly higher expression (*p* < 0.01) of PD-L1 than classical cells (Figure 3B).

SIRPalpha was also over-expressed by intermediate and non-classical cells in glioma patients compared to the control group (*p* < 0.05 and *p* < 0.01, respectively) (Figure 2H,I). The expression of SIRPalpha on classical monocytes is significantly lower (*p* < 0.05) than on non-classical (Figure 3E).

Regarding TIM-3, the slightly increased expression of TIM-3 in the non-classical monocytes compared to the control group was noted (Figure 2L). Furthermore, a comparison between monocyte subtypes revealed a drop in TIM-3 expression in non-classical vs. intermediate and classical monocytes (Figure 3C). Additionally, Monte Carlo Method was used to generate a larger sample to re-test the differences and the results are shown in Supplementary Table S1.

### 2.3. Intermediate Monocytes Were Skewed towards the Anti-Inflammatory Profile in Glioma Patients

**Figure 4:**
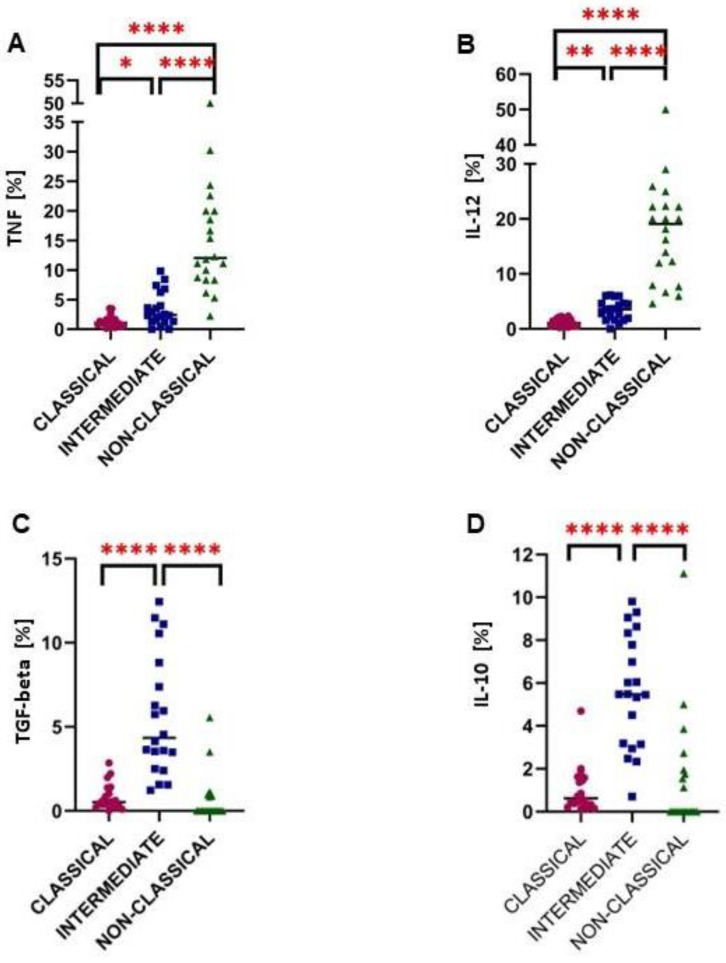
The comparison of the cytokine expression between classical, intermediate and non-classical monocytes. The pro-inflammatory molecules are noted in panels (**A**,**B**) (TNF and IL-12, respectively), whereas anti-inflammatory cytokines are distinguished in (**C**,**D**) graphs (TGF-beta and IL-10, respectively). * p < 0.05, ** *p* < 0.01, **** *p* < 0.0001. TNF, tumor necrosis factor; IL-12, interleukin 12; TGF-beta, transforming growth factor beta; IL-10, interleukin 10.

**Figure 5:**
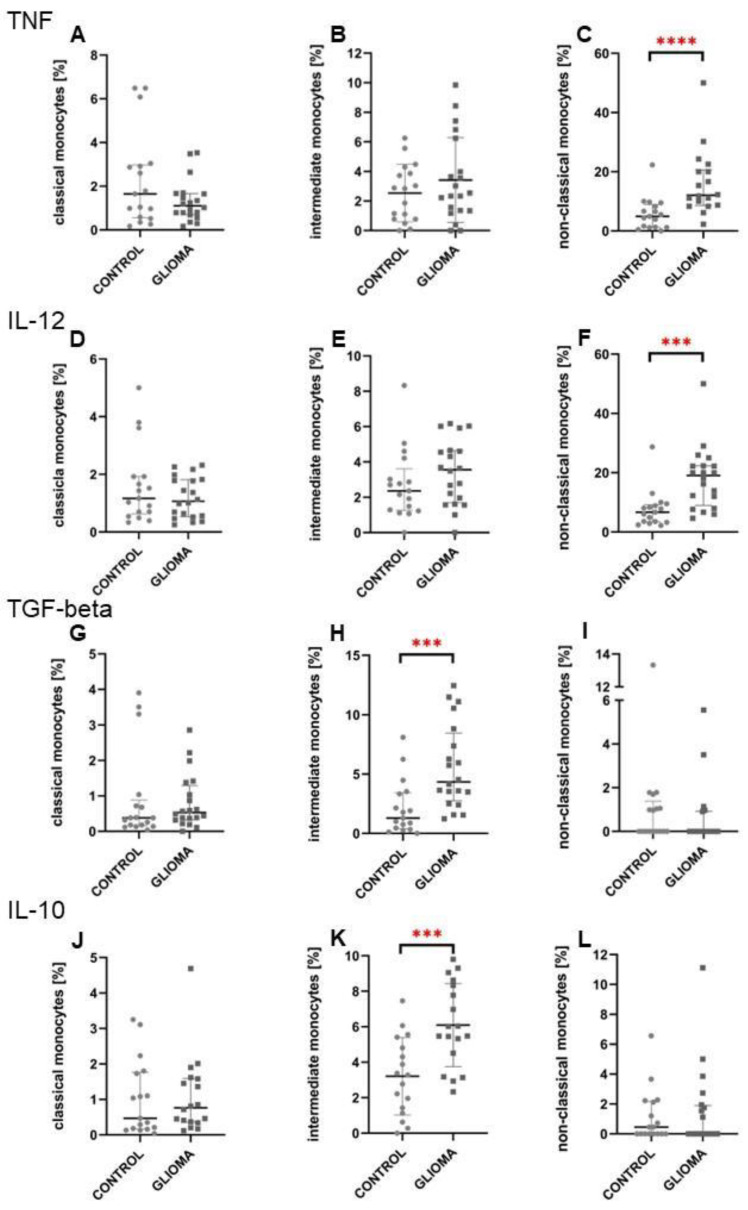
The results of cytokine molecules’ expression for three monocyte subpopulations compared to the healthy volunteers’ group. Both, pro-inflammatory TNF, IL-12 (**A**–**C**; **D**–**F**, correspondingly) and anti-inflammatory TGF-beta, IL-10 (**G**–**I**; **J**–**L**, respectively) were evaluated. Unpaired *t*-student was used for the statistical test, the results of which are presented in graph (**B**,**K**). In other cases U Mann-Whitney was used. *** *p* < 0.001, **** *p* < 0.0001. TNF, tumor necrosis factor; IL-12, interleukin 12; TGF-beta, transforming growth factor beta; IL-10, interleukin 10.

In the next step, the intracellular expression of cytokines in ex vivo conditions was analysed. Overall, non-classical monocytes had the highest levels of both TNF and IL-12 (Figure 4A,B); moreover, this was further up-regulated in glioma patients (Figure 5C,F). Quite the opposite was noted for intermediate monocytes that were characterised by the highest expression of anti-inflammatory IL-10 and TGF-beta (Figure 4C,D) with significant up-regulation among glioma patients (Figure 5H,K). The obtained results were confirmed by analysis on simulated data (Table S1).

### 2.4. Monocytes from Patients with the MGMT-Unmethylated Tumors Are Characterized by Higher Expression of IL-10

**Figure 6:**
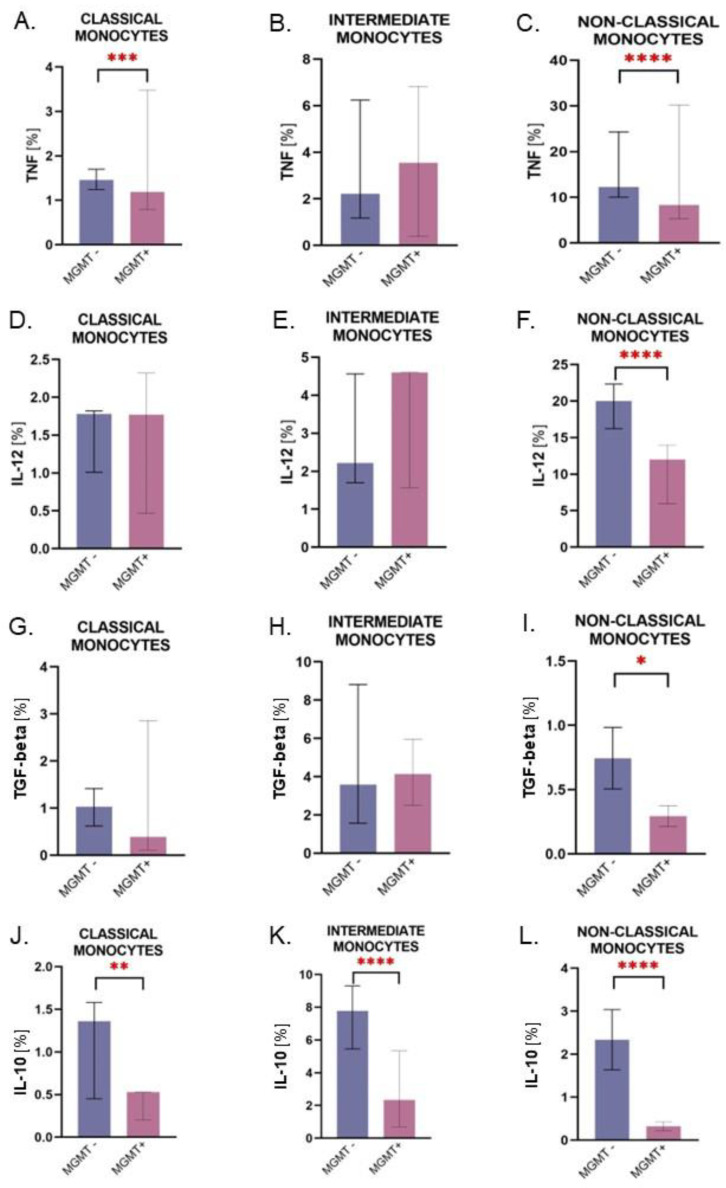
The figure shows differences between monocyte subpopulations’ cytokine expression in the context of *MGMT* gene promoter methylation status. Graphs (**A**–**F**) focus on pro-inflammatory molecules (TNF, IL-12), though graphs (**G**–**L**) visually present data on anti-inflammatory cytokines (TGF-beta, IL-10). **‘***MGMT*-‘ stands for *MGMT*-unmethylated tumor status, ‘*MGMT*+’ stands for *MGMT*-methylated tumor status; * *p* < 0.05, ** *p* < 0.01, *** *p* < 0.001, **** *p* < 0.0001.

A significant over-expression of TNF and IL-12 in non-classical monocytes from patients with MGMT-unmethylated status was noted (Figure 6C,F). Additionally, similar results were noted for TGF-beta expression (Figure 6K). Interestingly, IL-10 was over-expressed by all three monocyte subpopulations from patients with *MGMT*-unmethylated tumor compared to *MGMT*-methylated tumor (Figure 6G,H,I).

### 2.5. RNA Transcript Expression of PDCD1LG2 and HAVCR2 Gene Is Higher in GBMs’ Tissue Than in Healthy Control

**Figure 7:**
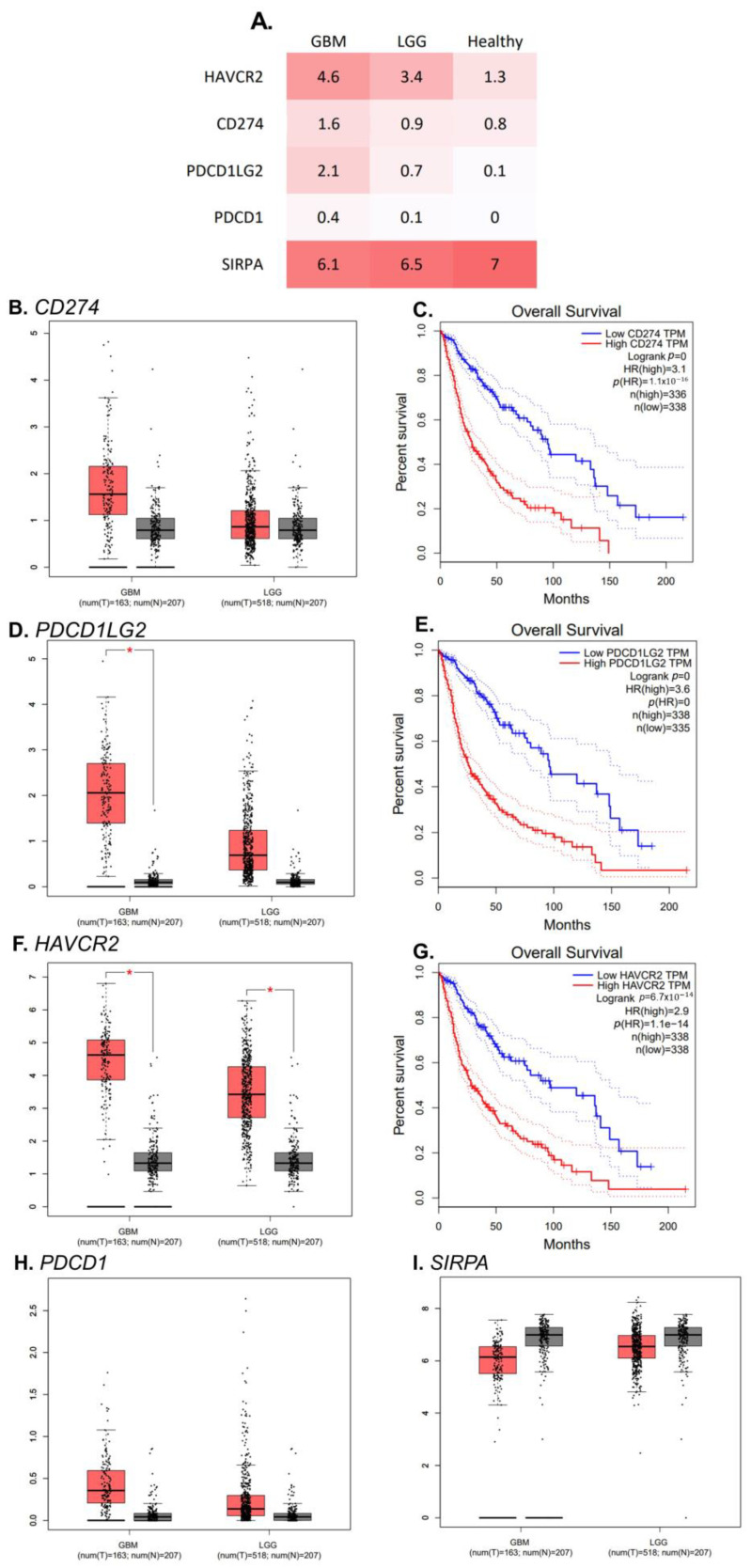
RNA transcript expression of *CD274*, *PDCD1LG2*, *HAVCR2, PDCD1, SIRPA* genes in GBM and LGG tissues and healthy control. Panel (**A**) summarizes differences in expression of analysed genes, it presents median Z-score. The lower TPM of each of the analysed genes is connected with more favorable overall survival when comparing gliomas (including LGG and GBM) vs. healthy tissue (**C**,**E**,**G**). The greatest benefit of low transcription is illustrated by Kaplan-Meier curve for *CD274* (**C**). Z-score of *PDCD1* gene in comparison between GBM and control showed significant difference (**D**). Additionaly, TIM-3 coding gene is characterised by a higher number of transcripts in both LGG and GBM (**F**). No significant differences were noted for *CD274, PDCD1* and *SIRPA* (**B**,**H**,**I**). The data described above were downloaded from The Cancer Genome Atlas (TCGA) database and Genotype-Tissue Expression (GTEx). The asterisk symbol (*) in the figure indicates a statistically significant difference between two compared groups. * *p* < 0.05 *HAVCR2*- Hepatitis A virus cellular receptor 2, TIM-3 gene; *CD274*- PD-L1 coding gene; *PDCD1LG2*-PD-L2 coding gene; *PDCD1*-PD-1 coding gene; *SIRPA*- SIRPalpha coding gene; TPM- transcript per million; LGG-low grade glioma (WHO grading I and II); GBM-glioblastoma (WHO grading IV).

Based on the data downloaded from The Cancer Genome Atlas (TCGA) database and Genotype-Tissue Expression (GTEx), significant overexpression of *PDCD1LG2* (Figure 7D) and for *HAVCR2* was noted within tumour tissue (Figure 7F). In general, gliomas had lower transcript expression of *SIRPA* (gene encoding SIRPalpha) (Figure 7I) and higher of PD-L1 (*CD274*), PD-L2 (*PDCD1LG2*), TIM-3 (*HAVCR2*), PD-1 (*PDCD1*) (Figure 7B,D,F,H, respectively). Lower transcript per million (TPM) of *CD274*, *PDCD1LG2*, *HAVCR2* is connected with better overall survival (Figure 7C,E,G).

## 3. Discussion

The question about the origin of each subpopulation of monocytes remains unanswered. It is still unclear whether monocyte subpopulations are the final steps in other common precursor differentiation pathways; they may represent the successive stages of maturation of monocytes derived from the same progenitor cell, with intermediate monocytes representing a phenotypically functional transient form between classical and non-classical monocytes [27]. Monocytes are beginning to be seen as cells that play an important role not only in physiological processes but also in pathological conditions, e.g., cancer or autoimmune diseases [28,29].

The importance of monocyte subpopulations in the pathogenesis of gliomas is still unknown. Recent studies have shown that monocytes could carry brain-specific proteins, which suggests that wider knowledge of their biology could be helpful in the diagnosis of gliomas and other brain tumors [25]. This discovery is a milestone in the evaluation of the potential role of monocytes in the pathogenesis of brain tumours.

The present study showed that the classical monocytes dominate among all monocytes in both healthy and glioma patients. In turn, the least numerous were non-classical monocytes; additionally, we observed their reduced percentage in gliomas compared to healthy people. Likewise, decreased percentage of non-classical monocytes was also observed in other central nervous system disorders. Waschbisch et al. noted reduced percentages of non-classical monocytes in the blood of untreated relapsing-remitting multiple sclerosis patients [30]. Interestingly, only the percentage of intermediate monocytes is higher in glioma patients compared to the control group. This data is similar to Prat et al.’s results [31] who observed an increased frequency of intermediate monocytes in patients with ovarian cancer, but Kwiecień et al. also observed this tendency in patients with non-small cell lung cancer [32]. The current, interaction-based study of immune checkpoints in glioma immunology has so far focused on the expression of PD-1 on T cells [33,34]. Besides T lymphocytes, antigen-presenting cells (APCs) also express PD-1, but the role of PD-1-positive APCs in glioma biology is still under investigation. The expression of PD-1 on myeloid cells may contribute to the weakening of their antitumor nature [35]. A higher level of PD-1+ monocytes in hepatocellular carcinoma patients suggests a higher activation of these cells within tumor environment [36]. On the contrary, PD-1+ monocytes in hepatocellular carcinoma patients have a lower potential to support cytotoxic T cell activity. This suggests that the PD-1/PD-L1 pathway suppresses antitumor immunity not only by suppressing signalling in T cells but also by inhibiting antigen presentation by monocytes [35,36]. Intermediate monocytes are a subset of monocytes with high properties to antigen presentation compared to classical and non-classical [37]. We observed significantly higher expression of PD-1 on intermediate monocytes in glioma patients compared to healthy controls. Possibly, the increased expression of PD-1 on intermediate monocytes can impair their antigen presentation capacity and thus lower the activation of antitumor immunity. Moreover, we reported a noticeable drop in PD-1 expression in non-classical monocytes, but significantly higher expression of PD-L1 in intermediate and non-classical monocytes compared to classical monocytes. Probably, the subpopulations of monocytes expressing PD-L1 in the periphery induce T cell anergy.

SIRPalpha is a negative innate checkpoint molecule [38]. Monocytes expressing SIRPalpha can phagocyte tumor cells with low levels of CD47 on their surface; the interaction CD47-SIRPalpha protects normal cells from this fate [39]. Indeed, the high expression of CD47 on tumor cells is associated with poor prognosis [40]. In our study, the overexpression of SIRPalpha in monocytes was observed in glioma patients. On the other hand, the classical monocytes had lower expression of SIRPalpha than intermediate and non-classical cells which suggests that classical monocytes are less able to interact with CD47 and thus less sensitive to induce an antiphagocytic signal in glioma patients. Li Xuenn et al. [41] observed that the percentage of TIM-3^+^ CD14^+^ monocytes was significantly higher in glioma patients compared to healthy people. We observed higher expression of TIM-3 in classical monocytes, but among intermediate and non-classical monocytes the percentage of TIM-3+ cells was lower. Likewise, TIM-3 has inhibitory properties for human monocytes [42]. The cytokine profile of each monocyte subset remains a matter of dispute [9,28,43]. In a mouse model of glioma, in which cells from the glioma line GL261 were implanted into C57BL6.CCR2^RFP/WT^ mice, the level of expression of genes associated with pro- and anti-inflammatory cytokines in monocytes and glioma macrophages was assessed. Monocytes display reduced gene expression for IFN-γ as well as for IL-4 and IL-10 when compared to brain macrophages. This suggests that both macrophages in glioma and monocytes in the periphery have different cytokine profiles [44]. We focused on the ability of monocyte subpopulations to produce TNF, IL-12, TGF-beta and IL-10 in the peripheral blood directly ex vivo. The proinflammatory cytokines (TNF, IL-12) were dominantly expressed by non-classical monocytes in glioma patients. This suggests that non-classical monocytes play an important role in antitumor response. Similar results were observed in research on haematological cancer including chronic lymphocytic leukemia or myelodysplastic syndrome [45,46]. Interestingly, our data suggests that the intermediate monocytes have higher intracellular expression of IL-10 and TGF-beta compared to classical and non-classical monocytes. Mukherjee et al. also suggested that intermediate monocytes mainly produce IL-10, while nonclassical monocytes secrete TNF and IL-1β [47]. The role of monocytes producing IL-10 and TGF-beta in cancer is ambiguous. First, they have immunosuppressive properties so they can promote tumor growth. On the other hand, these cells inhibit chronic inflammation and limit the development of the tumor [48,49]. Ochocka et al. presented transcriptional characteristics of monocytes/macrophage subpopulations, microglia and CNS border-associated macrophages in glioma mice model with GL261 cell line using single-cell RNA sequencing (scRNA-seq) [50]. They noted subpopulations of monocytes/macrophages in mice expressing high levels of CD274 and suggested that these cells play an immunoregulatory role in gliomas microenvironment [50]. Based on TCGA and CGGA datasets, the importance of *CD274* expression in glioma cells in infiltration of macrophades has been suggested [51].

Schaafsma et al. reported that immune-regulated-genes (IRG), including *HAVCR2*, *CD276*, *CD274,* are overexpressed on LGGs and GBMs cells [52]. Moreover, they are connected with poor prognosis and shorter overall survival [52]. Zhang et al. observed that IDO1, PD-L1 (*CD274*), PD-L2 (*PDCD1LG2*), TIM-3 (*HAVCR2*), PD-1 (*PDCD1*), LAG3, ICOS, and CD27 were highly expressed in the high-risk compared to the low-risk gliomas, which suggests that high risk glioma groups can be more sensitive to immunotherapy [53]. Vlaminck et al. used scRNA-seq and CITE-seq (cellular indexing of transcriptomes and epitopes sequencing) datasets of human and mouse GBM and showed an elevated expression of SIRPalpha in monocytes, TAMs and dendritic cell subsets [54]. Moreover, they generated nanobodies against SIRPα, passing the blood brain barrier which can target SIRPalpha+ GBM-infiltrating myeloid cells [54]. *MGMT* methylation status is a useful marker in the prognosis of glioblastoma. This gene may be silenced by methylation of its promoter and as a result the repair of DNA becomes hindered [55]. *MGMT* gene promoter methylation is associated with longer survival in patients with GBM [7]. Our results showed that all three monocyte subpopulations presented higher expression of IL-10 from patients without *MGMT* methylation in tumor cells. In their study, Kmiecik et.al. focused on the immunologic profile of glioblastoma microenvironment along with systemic characterization of immune cells [56]. The significantly eleveted level of immunosupressive IL-10 was noted in patients’ plasma when compared to control group [56]. Interestingly, the Chekenay group used Bortezomib pretreatment with TMZ, to increase the *MGMT*-methylation within tumor tissue [57]. They observed that patients with positive treatment outcomes, which is connected to lower methylation *MGMT* levels, have lower IL-10 in plasma [57]. Zhao et al., on the basis the TCGA and CGGA (Chinese Glioma Genome Atlas) cohorts, showed that the high-risk group gliomas are associated with up-expression of *CD274,CD276* and *CD44* [58]. In our analysis using TCGA, RNA sequencing data observed lower RNA transcript expression of *PDCD1LG2,* and *HAVCR2* gene is higher in GBMs’ tissue than in healthy control. Ding at al. using the CGGA database found that the overexpression of immune checkpoint genes: *CTLA4, CD274, HAVCR2, PDCD1, PDCD1LG2, SIGLEC15* and *TIGIT* was significant with poor survival [59]. We found that lower transcript of *CD274*, *PDCD1LG2*, *HAVCR2* is connected with better patients’ overall survival.

## 4. Materials and Methods

### 4.1. Study Group

The study group consisted of 24 patients diagnosed with glioma (WHO grade II, III and IV) confirmed by histopathological examination. Samples of PB were collected in EDTA-coated tubes from the patients admitted for tumor surgery at the Department of Neurosurgery and Paediatric Neurosurgery of the Medical University of Lublin, Poland. The clinical characteristics of glioma patients are summarized in Table 1. Prior to the procedure, patients provided written informed consent, in accordance with Bioethical Committee-approved protocols (Bioethical Committee at the Medical University of Lublin, KE-0254/28/02/2022). The control group included 24 healthy volunteers, matched in terms of age and sex. Exclusion criteria: autoimmune disease, glucocorticoid intake in the previous 4 weeks and oncological history.

### 4.2. Flow Cytometry

#### 4.2.1. Surface Staining

**Figure 8:**
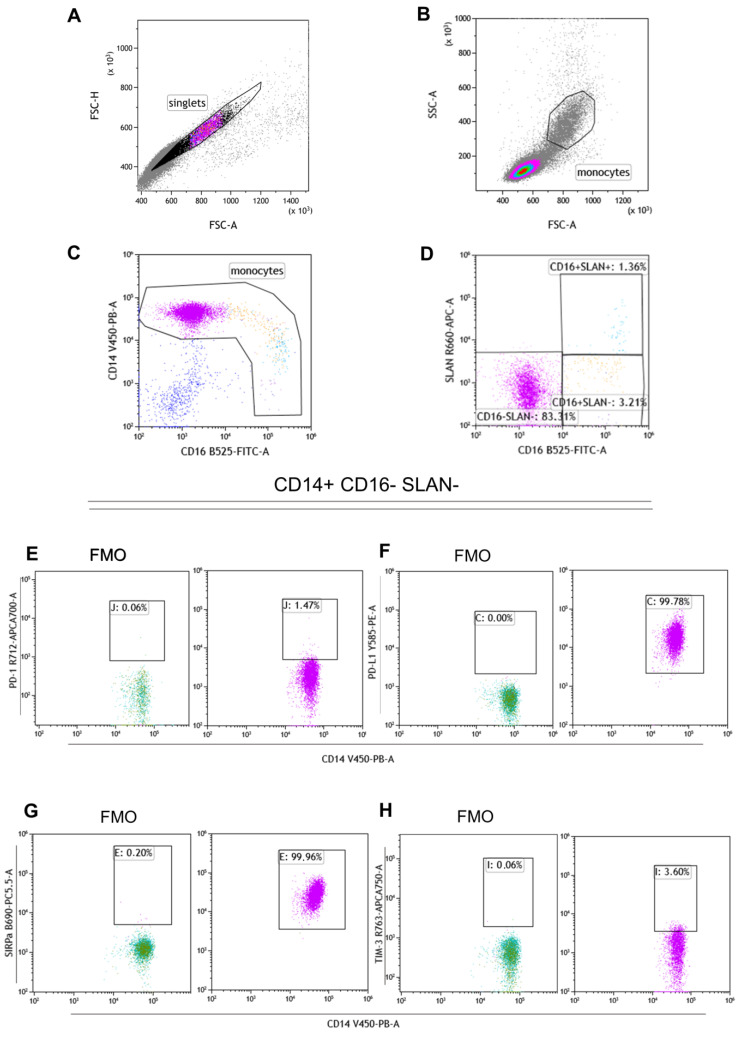
The gating strategy for monocyte subpopulations is demonstrated in (**A**–**D**) dot plots. Doublets elimination and setting up gate covering on singles population is presented in panel (**A**). Therefore, FSC-A vs. SSC-A gating was used for monocyte selection (**B**). For a more accurate determination of the monocyte population, assessment of CD14 V450 vs. CD16 FITC was used (**C**). The gating from the panel (**D**) CD16 FITC vs. SLAN APC was used to identify classical (CD14^+^ CD16^−^ SLAN^−^), intermediate (CD14^dim^ CD16^+^ SLAN^−^) and non-classical (CD14^low/−^ CD16^+^ SLAN^+^) monocytes. The assessment of immune checkpoint molecules based on fluorescence-minus-one (FMO) control for classical monocytes (CD14^+^ CD16^−^ SLAN^−^) is shown in dot plots (**E**–**H**).

Peripheral blood mononuclear cells (PBMCs) were isolated from whole blood by density-gradient centrifugation (Lymphocyte Separation Medium 1077, PromoCell, #C-44010, Heidelberg, Germany). PBMCs were stained with V450 anti-CD14 (#564406, clone: MφP9, BD Biosciences, Franklin Lakes, NJ, USA), FITC anti-CD16 (#555406, clone: 3G8, BD Biosciences), and after 10 min of incubation at room temperature (20 °C) in darkness APC anti-SLAN (M-DC8) (#130-119-865, clone: DD–1, Miltenyi Biotec, Bergisch Gladbach, NRW, Germany) was also added for the next 10 min of incubation at 4 °C. The following immune checkpoints monoclonal antibodies (mAb) were used: Alexa Fluor 700 anti-PD-1 (#329952, clone: EH12.2H7, Biolegend, San Diego, CA, USA), PE anti-PD-L1 (#557924, clone: MIH1, BD), APC-Cy7 anti-TIM-3 (#345026, clone F38-2E2, Biolegend), PerCP-eFluor 710 anti-CD172a (SIRPalpha) (#46-1729-42, clone 15-414, Invitrogen, Carlsbad, CA, USA). Cells were also incubated with chemokine receptors mAb: PE-Cy7 anti-CCR2 (#357212, clone: K036C2, Biolegend), BV605 anti-CCR5 (#563379, clone: 2D7/CCR5, BD Biosciences), APC-Cy7 anti-CX3CR1 (#341616, clone 2A9-1, Biolegend). Then cells were washed with phosphate-buffered saline (PBS) (#ECB4004L, Euroclone, Pero MI Italy) and analyzed. Based on CD14, CD16 and SLAN expression, monocytes classical, intermediate and non-classical subpopulations were identified. The gating strategy of monocyte subpopulations is presented in Figure 8A–D.

#### 4.2.2. Intracellular Staining

Staining with anti-CD14, anti-CD16 and anti-SLAN was performed as described in 4.2.1. Next, PBMCs were fixed in 1% paraformaldehyde and washed twice with Perm/Wash (# 554714, BD Biosciences) before proceeding to intracellular staining with the following cytokines mAb: BV 786 anti-IL-10 (#564049, clone JES3-9D7, BD Biosciences) PE anti-TGF-beta (#562339, clone TW4-9E7, BD Biosciences) PerCP anti-IL-12 (#MA5-23622, clone 27537, Invitrogen) BV 510 anti-TNF (#502950, clone: MAb11, Biolegend). Afterwards, probes were incubated at 4 °C in the darkness for one hour and then washed with PBS. Sample acquisition was performed with CytoFlex LX (Beckman Coulter, Brea, CA, USA) and Kaluza (Beckman Coulter) was used for flow cytometry data analysis. If the expression of the given molecule exceeded 95%, the mean fluorescence intensity (MFI) was evaluated. MFI implicitly suggests antigen expression [60]. The gating strategies for intracellular stainings are presented in Figure 8E–H.

### 4.3. Single-Cell RNA-Seq Analysis

The analysis of the single-cell RNA-seq was conducted using the GEPIA tool [61]. GEPIA used RNA-seq transcriptional profiles of various tumors downloaded from The Cancer Genome Atlas (TCGA) dataset [62]; the data on healthy tissues is from The Common Fund’s Genotype-Tissue Expression (GTEx) [63]. Transcriptional profiles of low-grade gliomas (LGGs) and glioblastomas were compared with healthy central nervous system tissue. Focusing on GBM and LGGs, the following genes were evaluated: *HAVCR2, CD274, PDCD1LG2, PDCD1, SIRPA*. The median of Z-scores for individual mRNAs are presented as boxplots and summarized as a heatmap. The Kaplan-Meier Survival Curves were prepared for LGGs and GBMs as one group.

### 4.4. Statistical Analysis

Statistical analysis was performed with GraphPad Prism 9 (GraphPad Software, San Diego, CA, USA) and Statistica software (version 13.3. StatSoft, Tulsa, OK, USA). Data distribution was analyzed with the D’Agostino & Pearson test. The U Mann-Whitney test or the unpaired *t*-test were used depending on the normality of data distribution. Median and interquartile range were determined for data with other than normal distribution, otherwise mean and standard deviation were utilized. *p* values of <0.05 were considered statistically significant. Based on the obtained data, the Monte Carlo Method was used to simulate 100 records and, due to the non-normal distribution, the U Mann-Whitney tests were conducted. The Kruskal-Wallis test without Dunn correction was performed to compare the expression of selected molecules by different subpopulations of monocytes. Moreover, to compare the expression of studied cytokine molecules on monocytes from patients with or without *MGMT* gene promoter methylation, the multiple probability simulation (Monte Carlo Method) was used with a number of generated results equal to 100. Thereafter, due to the non-Gaussian distribution of obtained data, results were analyzed with U Mann-Whitney tests.

## 5. Conclusions

The phenotypical and functional role of subpopulations of monocytes in the peripheral blood of glioma patients is ambiguous. The over-expression of PD-L1 and SIRPalpha along with lower expression of TIM-3 on the surface of non-classical monocytes and high expression of TNF and IL-12 suggests their dual role in promoting glioma-induced immunosuppression on the periphery and in stimulating the antitumor response. Monocytes could be an additional factor that should be taken into account when considering the effectiveness of potential immunotherapy.

## Figures and Tables

**Table 1:** Clinical characteristics of the cohort group.

Characteristics	Glioma Patients *n* = 24	Healthy Volunteers *n* = 24
Gender:		
Male [*n*]	18	12
Female [*n*]	6	12
Age		
Mean [yr]	10	56
Min [yr]	22	44
Max [yr]	82	78
WHO grade		
I [%]	0	
II [%]	8.33	
III [%]	20.83	
IV [%]	62.5	
Glioblastoma multiforme [*n*]	16	
IDH status:		
Mutant [*n*]	4	
Wildtype [*n*]	20	
MGMT status (among Glioblastoma multiforme; *n* = 16)		
MGMT-methylated [*n*]	4	
MGMT-unmethylated [*n*]	11	
Unknown status [*n*]	1	
Monocytes (before surgery)		
Mean [K/µL]	0.475	

IDH, Isocitrate dehydrogenase; MGMT, O-6-methylguanine-DNA methyl-transferase.

## Data Availability

The data presented in this study are available within the article. Other data that support the findings of this study are available upon request from the corresponding author.

## Supplementary Materials

The following supporting information can be downloaded at: https://www.mdpi.com/article/10.3390/ijms24031879/s1.
