# Supplementary material for: Pro- vs. Anti-Inflammatory Features of Monocyte Subsets in Glioma Patients
Source: Int J Mol Sci. 2023 Jan 18;24(3):1879. doi: 10.3390/ijms24031879 (PMC9915868; doi:10.3390/ijms24031879)
Supplement: Supplementary file 1 [file ijms-24-01879-s001.zip › ijms-2111324-supplementary.pdf]

Table S1: ***P*-values for the data obtained by the multiple probability simulation in glioma patients' compared to a healthy volunteer group.**

	***p*-value**		
	**Classical Monocytes**	**Intermediate Monocytes**	**Non-classical monocytes**
**PD-1 [%]**	-	<0.0001	-
**PD-L1 [%]**	<0.0001	<0.0001	<0.0001
**PD-L1 MFI**	0.8822	<0.0001	<0.0001
**SIRPalpha [%]**	<0.0001	<0.0001	0.0842
**SIRPalpha MFI**	<0.0001	0.0001	<0.0001
**IL-12 [%]**	0.8706	0.0014	<0.0001
**TNF [%]**	0.0554	0.003	<0.0001
**IL-10 [%]**	0.4675	<0.0001	0.6493
**TGF-beta [%]**	0.0008	<0.0001	0.0005

To confirm the significance of the obtained results, the Monte Carlo Method was used to simulate the data (n=100) based on the results (n=24) which showed statistical significance in comparison between the expression of immune checkpoint and cytokine molecules expression in glioma patients' compared to a healthy volunteer group. '-' symbolize the absence of statistical significance when comparing 24 original results to the control.

PD-1, programmed cell death 1 protein; PD-L1, programmed cell death 1 ligand, MFI, mean fluorescence intensity;SIRPalpha, signal-regulatory protein alpha; TNF, tumor necrosis factor; TGF-beta, transforming growth factor beta
